# Césarienne à Lubumbashi, République Démocratique du Congo II: facteurs de risque de mortalité maternelle et périnatale

**DOI:** 10.11604/pamj.2017.26.208.12148

**Published:** 2017-04-17

**Authors:** Xavier Kinenkinda, Olivier Mukuku, Faustin Chenge, Prosper Kakudji, Peter Banzulu, Jean-Baptiste Kakoma, Justin Kizonde

**Affiliations:** 1 Département de Gynécologie-Obstétrique, Faculté de Médecine, Université de Lubumbashi, République Démocratique du Congo; 2 Institut Supérieur des Techniques Médicales de Lubumbashi, République Démocratique du Congo; 3 Département de Gynécologie-Obstétrique, Faculté de Médecine, Université de Kinshasa, République Démocratique du Congo

**Keywords:** Césarienne, mortalité maternelle, mortalité périnatale, facteurs de risque, Lubumbashi, Cesarean section, maternal mortality, perinatal mortality, risk factors, Lubumbashi

## Abstract

**Introduction:**

L’objectif était d’analyser les facteurs de risque de mortalité maternelle et périnatale de la césarienne à Lubumbashi, République Démocratique du Congo (RDC).

**Méthodes:**

Étude multicentrique de 3643 césariennes réalisées entre le 1^er^ janvier 2009 et le 31 décembre 2013 sur un total de 34199 accouchements dans cinq formations hospitalières de référence à Lubumbashi (RDC). Les données sociodémographiques, les indications, l’environnement obstétrical et la morbi-mortalité maternelles et périnatales ont été analysés au logiciel Epi Info 2011. Les fréquences calculées sont exprimées en pourcentage et les moyennes avec leurs écart-types. Le test de Chi-carré et le test exact de Fisher lorsque recommandés ont été utilisés pour la comparaison des fréquences. L’odds ratio a été calculé avec l’intervalle de confiance de 95% de Cornfield grâce à un modèle de régression logistique pour déterminer la puissance de facteurs de risque. Le seuil de signification a été fixé à p < 0,05.

**Résultats:**

La fréquence de la césarienne était de 10,65%. L'âge moyen des césarisées était de 28,83±6,8 ans (extrêmes: 14 et 49 ans). La parité variait de 1 à 16 avec une moyenne de 2,6. De ces opérées, une sur neuf (10,9%) était porteuse d’un utérus cicatriciel de césarienne antérieure et 22,3% étaient des évacuées obstétricales. Les taux de létalité maternelle et périnatale étaient respectivement de 1,4% et 7,07% lors de la césarienne. L’analyse des facteurs de risque montre que la grande multiparité (≥5), l’absence de surveillance de la grossesse, le caractère urgent de l’indication opératoire influent significativement sur la mortalité maternelle. A ces facteurs s’ajoutent pour la mortalité périnatale l’âge maternel avancé (> 35 ans), l’évacuation comme mode d’admission et l’immaturité fœtale.

**Conclusion:**

Cette étude montre que la césarienne dans nos conditions de travail est couplée à une forte mortalité maternelle et périnatale. Les facteurs de risque identifiés sont en grande partie évitables, surtout à tort ou à raison imputés à l’opération masquant ipso facto les circonstances souvent irrationnelles de sa pratique.

**Introduction:**

The objective was to analyze risk factors for maternal and perinatal mortality among women undergoing cesarean section in Lubumbashi, Democratic Republic of Congo (DRC).

**Methods:**

We conducted a multicenter study of 3643 women undergoing cesarean sections between 1 January 2009 and 31 December 2013 out of 34199 women delivering in five general referral hospitals in Lubumbashi (DRC). Sociodemographic data, indications, obstetrical environment as well as maternal and perinatal morbi-mortality were analyzed using Epi Info 2011 software. Computed frequencies were expressed in percentage and mean values were expressed in terms of standard deviations. Chi-square test and Fisher’s exact test, when recommended, were used to compare frequencies. The odds ratio was calculated using Cornfield 95% confidence interval based on a logistic regression model in order to determine the strength of risk factors. Threshold significance level was set at p < 0.05.

**Results:**

The frequency of cesarean sections was 10.65%. The average age of women undergoing cesarean section was 28.83 ± 6.8 years (with a range from 14 to 49 years). Parity ranged from 1 to 16 with an average of 2.6. 1 out of 9 (10.9%) women undergoing cesarean section were patients with previous caesarean section uterine scar on the anterior wall of the uterus and 22.3% of women were patients with previous obstetric evaquation. Maternal and perinatal mortality rate was 1.4% and 7.07% during cesarean section respectively. The analysis of risk factors shows that the great multiparity (≥5), the absence of monitoring during pregnancy, the urgent nature of emergency surgery significantly affect maternal mortality. Other factors for perinatal mortality included advanced maternal age (>35 years), patients referral from one facility to another as a mode of admission and fetal immaturity.

**Conclusion:**

This study shows that cesarean section in our working condition is associated to a significant maternal and perinatal mortality. Identified risk factors are largely preventable, because they are rightly or wrongly ascribed to cesarean section glossing over, ipso facto, the often irrational circumstances of its practice.

## Introduction

L’état de santé des mères et celui de leurs enfants sont intimement liés et c’est sur eux que repose le développement de la société [1]. C’est pour cette raison que la mortalité maternelle et périnatale est un indicateur précieux de l’efficacité des soins obstétricaux et du degré de développement d’un pays [2-7]. L’Organisation Mondiale de la Santé (OMS) ne cesse de faire des efforts importants pour l’amélioration de la santé maternelle et infantile qui a enregistré des progrès, notamment en Afrique subsaharienne. Toutefois, dans cette région du monde, le risque pour une femme de mourir pendant la grossesse ou l'accouchement est encore très élevé, de 1 sur 39 contrairement aux pays développés, où ce risque de mortalité maternelle est devenu moindre, estimé à 1 sur 3700 [8]. La mortalité périnatale y est également élevée, supérieure à 21 décès pour 1000 naissances vivantes [9]. On trouve à la base de ce drame sanitaire la pauvreté, l’insuffisance des services de santé ou leur mauvaise répartition, certains facteurs ethnoculturels promoteurs de mariages précoces et les maternités nombreuses [10-15]. Dans la stratégie pour la maternité sans risque, la césarienne est un des moyens préconisés [16, 17]. Les progrès extraordinaires réalisés dans le domaine médical concernant les techniques opératoires et anesthésiques, la révolution considérable qu’a connue le nursing dans l’élevage des bébés nés fragiles et la surveillance postopératoire des mères, ainsi que le développement de la pharmacologie ont fait que, non seulement la fréquence de la césarienne a été élevée à des taux stupéfiants, mais aussi ses indications ont été fort diversifiées [18-22]. De nos jours, dans les pays développés, l’opération césarienne est devenue une intervention banale tant par la facilité avec laquelle elle est pratiquée que par la morbidité et la mortalité insignifiantes sur le couple mère-enfant [23]. En effet, la recrudescence de la césarienne dans ces pays s’est accompagnée d’un bénéfice proportionnel pour le couple mère-enfant. Aujourd’hui, avec des taux de césariennes variant entre 15 et 50% des accouchements, la mortalité maternelle y afférente y est inférieure à 0,03% et la mortalité périnatale presque nulle [8, 9, 23, 24]. Et pourtant, les pays en développement dont la République Démocratique du Congo (RDC), demeurent encore exclus de ces avancées scientifiques et technologiques. Les différentes statistiques rapportées à travers toute l’Afrique subsaharienne montrent que la césarienne reste une opération dangereuse pour le couple mère-enfant dans nos conditions de travail avec une forte morbidité et mortalité maternelles et périnatales [10, 11, 25-31]. Une étude menée à l’hôpital Sendwe à Lubumbashi (République Démocratique du Congo) avait trouvé des taux de létalité maternelle et périnatale spécifiques respectivement de 1,6% et 19,51% [10]. La présente étude s’est fixé comme objectif d’identifier et d’analyser les facteurs de risque de mortalité maternelle et périnatale liées à la césarienne à Lubumbashi, RDC. Une meilleure connaissance de ces facteurs de risque contribuera à rendre la césarienne moins dangereuse pour le couple mère-enfant par la mise au point d’une stratégie de prévention ou de contrôle de ces facteurs.

## Méthodes

L’étude s’est déroulée dans cinq formations hospitalières de référence de la ville de Lubumbashi (hôpital Gécamines/Sud, hôpital SNCC, Cliniques Universitaires, Centre médical Afia-Don Bosco et hôpital Jason Sendwe). Il s’agit d’une étude rétrospective descriptive et analytique des césariennes réalisées entre le 1er janvier 2009 et le 31 décembre 2013. Le recrutement a été exhaustif. Les registres d’accouchements, les partogrammes, les comptes rendus opératoires, et les fiches de nouveau-nés en néonatologie ont constitué la source d’information. Les paramètres étudiés étaient: l’âge maternel (< 20 ans = maternité précoce et > 35 ans = maternité tardive), la parité (nullipare = parité 0, paucipare = parité 1 à 2, multipare = parité 3 à 4 et grande multipare = parité 5 et plus), le statut matrimonial (femmes vivant seules et femmes vivant en union), les antécédents maternels (utérus cicatriciel de césarienne, hypertension artérielle, affection médico-chirurgicale générale et chirurgie gynécologique), le mode d’admission à la maternité (évacuation obstétricale = transfert d’une autre formation hospitalière), les consultations prénatales (suivies ou non), l’âge gestationnel (< 32 SA = grande immaturité, 32-36 SA = petite immaturité et ≥ 37 SA = grossesse à terme), les indications (maternelles, fœtales, annexielles ou mixtes), le type d’indications (élective ou non), le caractère d’indications (urgent ou non), le type de césariennes (segmentaire, corporéale; la laparotomie pour rupture utérine a été comptabilisée comme mode de terminaison pour mettre en exergue cette complication obstétricale), le type d’anesthésies (générale ou rachianesthésie), la notion de transfusions sanguines, le moment d’intervention (jour ou nuit), le poids du nouveau-né (< 2500 grammes = faible poids et ≥ 4000 grammes = macrosomie) et l'évolution maternelle et périnatale (survie ou décès). Les données recueillies ont été codifiées, saisies à l’aide du logiciel Microsoft Excel 2011 puis analysées à l’aide du logiciel Epi-Info 7.1.1. Les fréquences exprimées en pourcentage ont été calculées et leurs comparaisons effectuées grâce au test de Chi-carré corrigé de Yates ou le test exact de Fisher lorsque recommandés. L’odds ratio (OR) et ses intervalles de confiance à 95% (IC à 95%) ont été calculés pour évaluer la puissance des facteurs de risque. Le seuil de signification a été fixé à p < 0,05. Éthiquement, avant la récolte de données, les autorisations respectives des Médecins Directeurs des cinq formations hospitalières concernées et des comités provinciaux d’éthique avaient été obtenues après que nous ayons exposé les objectifs et la méthodologie de l’étude. Les données étaient collectées de manière anonyme. L’étude n’a pas présenté de bénéfice direct notamment lucratif pour les participants à l’étude.

## Résultats

### Fréquence de la césarienne et caractéristiques socio-démographiques et cliniques des opérées

Durant la période d’étude, les maternités ont enregistré 34199 accouchements et 34980 naissances. Parmi ces accouchements, 3643 étaient des césariennes (10,65%) desquelles sont issus 3828 nouveau-nés. L’âge maternel moyen était de 28,83±6,79 ans (extrêmes: 14 - 49 ans). Sept opérées sur dix avaient un âge compris entre 20 et 35 ans. La maternité précoce (âge ?20 ans) représentait 10,0% des accouchements. La parité moyenne était de 2,6±2,5 (extrêmes: 0 – 16). Plus de 30% des patientes étaient primipares et 92,1% d’entre elles s’étaient déclarées vivant en union. Environ onze pourcent des mères étaient porteuses d’un utérus cicatriciel de césarienne antérieure, 22,3% étaient des évacuées obstétricales et environ 20% des accouchées n’avaient pas bénéficié de consultations prénatales. S’agissant du terme de la grossesse, il a varié entre 22 et 47 SA autour d’une moyenne de 37,9±2,8 SA, 5% des césarisées l’ont été sur des grossesses d’âge inférieur à 32 SA. En ce qui concerne les indications de césariennes, dans la plupart des cas, il s’agissait d’une indication maternelle (35,9%), suivie des indications mixtes (26,7%); 60,6% des indications n’avaient pas de caractère urgent mais 51,4% des césariennes ont été réalisées dans un contexte d’urgence obstétricale. Presque toutes les opérations l’ont été sous anesthésie générale (95,0%); la transfusion sanguine était réalisée dans près d’un cas sur 10 (**Tableau 1**).

**Tableau 1:** distribution des opérées selon les caractéristiques sociodémographiques et cliniques

Variable	Effectif	Pourcentage
**Âge maternel (n=3475)**		
< 20 ans	348	10,0
20-35 ans	2509	72,2
> 35 ans	618	17,8
Moyenne (extrêmes)	28,8±6,8 ans (14 – 49 ans)	
**Parité (n=3439)**		
0	1042	30,3
1-2	968	28,1
3-4	627	18,2
≥ 5	802	23,3
Moyenne (extrêmes)	2,6±2,5 (0 – 16)	
**Statut matrimonial (n=3475)**		
En union	3202	92,1
Seule	273	7,9
**Antécédents médicaux et chirurgicaux (n=3537)**		
Aucun	2757	77,9
Utérus cicatriciel de césarienne	387	10,9
HTA	180	5,1
Affection médico-chirurgicale générale	177	5,0
Chirurgie gynécologique	36	1,1
**Consultations prénatales (n=3333)**		
Suivies	2800	84,0
Non suivies	533	16,0
**Évacuation obstétricale (n=3491)**		
Non	2711	77,7
Oui	780	22,3
**Âge gestationnel (n=3346)**		
Grande immaturité	163	4,9
Petite immaturité	1016	30,4
Grossesse à terme	2167	64,7
**Contenu utérin (n=3643)**		
Monofoetal	3450	94,7
Gémellaire	184	5,1
Triplet	9	0,2
**Etiologie d’indications (n=3325)**		
Maternelle	1193	35,9
Mixte	889	26,7
Fœtale	730	22,0
Annexielle	513	15,4
**Type d’indications (n=3325)**		
Non élective	1708	51,4
Élective	1617	48,6
**Caractère d’indications (n=3325)**		
Non urgente	2016	60,6
Urgente	1309	39,4
**Type d’opérations (n=3397)**		
CS segmentaire	3096	91,1
CS corporéale	187	5,5
Lap-rupture utérine*	114	3,4
**Type d’anesthésie (n=3542)**		
Générale	3366	95,0
Rachianesthésie	176	5,0
**Moment d'intervention (n=3291)**		
Jour	1983	60,3
Nuit	1308	39,7

CS : césarienne ; *laparotomie

### Morbidité et mortalité maternelles et périnatales

La fréquence des complications maternelles per et postopératoires est de 11,6% (415 sur 3586); elle était dominée par l’hémorragie pathologique rencontrée dans 219 cas (52,77%). La morbidité néonatale traduite par les différentes complications observées en période néonatale précoce s’élevait à 28,6% dont 78,2% des cas de détresse respiratoire. L’on a enregistré 51 décès maternels sur les 3636 opérées (1,4%) et 249 décès périnatals sur les 3520 nouveau-nés (7,1%) dont l’évolution avait été précisée (**Tableau 2**).

**Tableau 2:** morbi-mortalité maternelle et périnatale

Variable	Effectif	Pourcentage
**Complications maternelles (n=3586)**		
Aucune complication	3171	88,4
Complications hémorragiques	219	6,1
Complications anesthésiques	56	1,6
Complications digestives et urinaires	43	1,2
Infections pariétales et endométriales	43	1,2
Infections respiratoires, affections cardio-vasculaires	8	0,2
Septicémie	6	0,2
Autres	40	1,1
**Évolution Maternelle (n=3636)**		
Survie	3585	98,6
Décès	51	1,4
**Complications néonatales (n=3377)**		
Aucune	2412	71,4
Détresse respiratoire	755	22,4
Traumatismes	54	1,6
Infections	33	1,0
Anémie	25	0,7
Autres	98	2,9
**Évolution périnatale (n=3520)**		
Survie	3271	92,9
Décès	249	7,1

### Mortalité maternelle et facteurs de risque

#### Mortalité maternelle et facteurs socio-démographiques, cliniques et obstétricaux

Parmi les 50 décès maternels sur 3432 opérées pour lesquelles la parité avait été précisée, 18 avaient concerné les grandes multipares (n=800; 23%) contre 32 sur les 2632 opérées avec une parité inférieure à 5 (12%) exprimant un risque létal multiplié par près de 2 (OR=1,87 (1,04-3,35)). Il en est de même des opérées n’ayant pas suivi les CPN comparées à celles qui en ont bénéficié (13 sur 518 contre 33 sur 2765; OR=2,10 (1,09-4,02)). Par ailleurs, les mères opérées sur terrain d’HTA ainsi que celles dont l’âge gestationnel était inférieur à 32 SA ont présenté un risque létal significatif (OR=6,61 (1,16-67,64) et 2,84 (1,06-7,54)). Par contre, l’âge maternel, le statut matrimonial, le contenu utérin et même le mode d’admission ne semblent influer sur l’issue maternelle. Les détails sur la mortalité maternelle et les facteurs socio-démographiques, cliniques et obstétricaux sont présentés dans le Tableau 3.

**Tableau 3:** mortalité maternelle en fonction des caractéristiques sociodémographiques, cliniques et obstétricales

	Décès		Survie		OR [IC à 95%]
Variable	n	%	n	%	
**Âge maternel (n=3468)**	**48**	**1,4**	**3420**	**98,6**	
<20 ans	5	1,4	343	98,6	1,00
20-35 ans	34	1,4	2470	98,6	0,94 [0,36-2,43]
>35 ans	9	1,5	607	98,5	1,01 [0,33-3,05]
**Parité (n=3432)**	**50**	**1,5**	**3382**	**98,5**	
0	9	0,9	1032	99,1	1,00
1-2	15	1,6	951	98,4	1,80 [0,78-4,15]
3-4	8	1,3	617	98,7	1,48 [0,57-3,87]
≥5	18	2,3	782	97,8	2,63 [1,17-5,90]
**Statut matrimonial (n=3468)**	**50**	**1,4**	**3418**	**98,6**	
En union	44	1,4	3151	98,6	1,00
Seule	6	2,2	267	97,8	1,60 [0,67-3,81]
**Antécédents (n=3532)**	**51**	**1,4**	**3481**	**98,6**	
Aucun	39	1,4	2713	98,6	2,74 [0,71-23,74]
Utérus cicatriciel de césarienne	2	0,5	385	99,5	1,00
Hypertension artérielle	6	3,3	174	96,7	6,61 [1,16-67,64]
Affection médico-chirurgicale générale	4	2,3	173	97,7	4,43 [0,62-49,49]
Chirurgie gynécologique	0	0,0	36	100	0,00 [0,00-57,83]
**Consultations prénatales (n=3329)**	**46**	**1,4**	**3283**	**98,6**	
Suivies	33	1,2	2765	98,8	1,00
Non suivies	13	2,4	518	97,6	2,10 [1,09-4,02]
**Contenu utérin (n=3636)**	**51**	**1,4**	**3585**	**98,6**	
Monofœtal	50	1,5	3394	98,5	0,35 [0,00-2,09]
Multiple	1	0,5	191	99,5	1,00
**Évacuation obstétricale (n=3485)**	**51**	**1,5**	**3434**	**98,5**	
Oui	14	1,8	764	98,2	1,32 [0,71-2,45]
Non	37	1,4	2670	98,6	1,0
**Âge gestationnel (n=3341)**	**44**	**1,3**	**3297**	**98,7**	
Grande immaturité	5	3,1	157	96,9	2,84 [1,06-7,54]
Petite immaturité	15	1,5	999	98,5	1,33 [0,69-2,56]
Grossesse à terme	24	1,1	2141	98,9	1,00

### Mortalité maternelle et environnement chirurgical

28 décès (58,33% des cas) sont survenus chez des opérées en urgence (n=1307) signifiant un risque létal égal à 2,1% rattaché au caractère urgent de l’intervention contre 20 décès sur 201 opérées (0,99%) sans urgence (OR=2,18 (1,22-3,88)). Notons également que sur 288 opérées ayant nécessité une transfusion sanguine, l’on a répertorié 11 décès (3,8%) contre 40 (1,2%) sur 3305 opérées non transfusées (OR=3,24 (1,64-6,38)) alors que l’indication, le caractère électif ou non, le type d’opération, le type d’anesthésie pratiquée, ni le moment de sa réalisation n’ont présenté une quelconque corrélation avec l’évolution maternelle (Tableau 4).

**Tableau 4:** mortalité maternelle en fonction de l’environnement chirurgical

Variable	Décès		Survie		OR [IC à 95%]
	n	%	n	%	
**Indications (n=3319)**	**48**	**1,4**	**3271**	**98,6**	
Maternelle	21	1,8	1168	98,2	1,75 [0,80-3,85]
Fœtale	7	1,0	721	99,0	0,94 [0,35-2,56]
Annexielle	11	2,1	502	97,9	2,14 [0,88-5,20]
Mixte	9	1,0	880	99,0	1,00
**Type d’indications (n=3319)**	**48**	**1,4**	**3271**	**98,6**	
Élective	24	1,5	1591	98,5	1,05 [0,59-1,86]
Non élective	24	1,4	1680	98,6	1,00
**Caractère d’indications (n=3319)**	**48**	**1,4**	**3271**	**98,6**	
Urgente	28	2,1	1279	97,9	2,18 [1,22-3,88]
Non urgente	20	1,0	1992	99,0	1,00
**Type d’opérations (n=3390)**	**48**	**1,4**	**3342**	**98,6**	
CS segmentaire	40	1,3	3049	98,7	1,00
CS corporéale	4	2,1	183	97,9	1,66 [0,42-4,68]
Lap-Rupture utérine*	4	3,5	110	96,5	2,77 [0,70-7,86]
**Type d’anesthésies (n=3535)**	**48**	**1,4**	**3487**	**98,6**	
Générale	44	1,3	3316	98,7	1,00
Rachianesthésie	4	2,3	171	97,7	1,76 [0,45-4,92]
**Transfusion (n=3593)**	**51**	**1,4**	**3542**	**98,6**	
Oui	11	3,8	277	96,2	3,24 [1,64-6,38]
Non	40	1,2	3265	98,8	1,00
**Moment d'intervention (n=3285)**	**47**	**1,4**	**3238**	**98,6**	
Jour	24	1,2	1956	98,8	1,00
Nuit	23	1,8	1282	98,2	1,46 [0,82-2,60]

*laparotomie

### Mortalité périnatale et facteurs de risque

#### Mortalité périnatale et facteurs socio-démographiques, cliniques et obstétricaux<

L’on a enregistré 174 décès parmi les 2759 nouveau-nés des opérées de moins de 36 ans d’âge (6,3%) et 58 sur 604 nouveau-nés des mères d’âge supérieur à 35 ans (9,6%). Les nouveau-nés des opérées ayant une parité inférieure à 5 ont présenté un taux de décès équivalant à 5,9% en comparaison avec 8,6% pour ceux nés des opérées ayant une parité d’au moins 5. La comparaison du risque létal en rapport avec l’âge maternel et la parité montre une différence statistique significative en défaveur des nouveau-nés des opérées plus âgées (OR=1,57 (1,15-2,15)) et des grandes multipares (OR=1,39 (1,03-1,88)). De même, les nouveau-nés des opérées n’ayant pas suivi les consultations prénatales, ceux des évacuées sanitaires et les prématurés ainsi que les nouveau-nés pesant moins de 2500 grammes ont présenté un taux de décès multiplié par 2. Les nouveau-nés des mères avec antécédent d’utérus cicatriciel n’avaient présenté un risque létal (5,3%) non significatif comparés à ceux dont les mères étaient hypertendues (8,6%; OR=2,97 (1,06-8,33)), ceux des mères avec antécédent de chirurgie gynécologique (14,3%; OR= 5,23 (1,42-19,19)) et ceux nés des mères sans antécédent (7,4%; OR=2,49 (1,01-6,16)). Par contre, le statut matrimonial ne semble jouer aucune influence sur l’issue périnatale (Tableau 5).

**Tableau 5:** mortalité périnatale en fonction de caractéristiques sociodémographiques, cliniques et obstétricales

	Décès		Survie		OR [IC à 95%]
Variable	n	%	n	%	
**Âge maternel (n=3363)**	**232**	**6,9**	**3131**	**93,1**	
<20 ans	18	5,3	322	94,7	1,00
20-35 ans	156	6,4	2263	93,6	1,23 [0,74-2,03]
>35 ans	58	9,6	546	90,4	1,90 [1,10-3,28]
**Parité (n=3325)**	**226**	**6,8**	**3099**	**93,2**	
0	60	5,8	981	94,2	1,00 [0,68-1,46]
1-2	53	5,7	869	94,3	1,00
3-4	49	8,0	565	92	1,42 [0,95-2,12]
≥5	64	8,6	684	91,4	1,53 [1,05-2,23]
**Statut matrimonial (n=3356)**	**235**	**7**	**3121**	**93**	
En union	217	7,0	2877	93,0	1,02 [0,62-1,68]
Seule	18	6,9	244	93,1	1,00
**Antécédents (n=3252)**	**230**	**7,1**	**3022**	**92,9**	
Aucun	185	7,4	2324	92,6	2,49 [1,01-6,16]
Utérus cicatriciel de césarienne	19	5,3	342	94,7	1,74 [0,63-4,75]
Hypertension artérielle	16	8,6	169	91,4	2,97 [1,06-8,30]
Affection médico-chirurgicale générale	5	3,1	157	96,9	1,00
Chirurgie gynécologique	5	14,3	30	85,7	5,23 [1,42-19,19]
**Consultations prénatales (n=3248)**	**220**	**6,8**	**3028**	**93,2**	
Suivies	156	5,7	2594	94,3	1,00
Non suivies	64	12,9	434	87,1	2,45 [1,80-3,33]
**Contenu utérin (n=3520)**	**249**	**7,1**	**3271**	**92,9**	
Monofœtal	219	6,9	2952	93,1	1,00
Multiple	30	8,6	319	91,4	1,26 [0,85-1,88]
**Évacuation obstétricale (n=3361)**	**237**	**7,1**	**3124**	**92,9**	
Oui	70	9,5	665	90,5	1,55 [1,15-2,07]
Non	167	6,4	2459	93,6	1,00
**Âge gestationnel (n=3243)**	**231**	**7,1**	**3012**	**92,9**	
Grand prématuré	34	24,6	104	75,4	5,44 [3,54-8,36]
Petit prématuré	77	7,8	908	92,2	1,41 [1,05-1,90]
Nouveau-né à terme	120	5,7	2000	94,3	1,00
**Poids de naissance (n=3299)**	**230**	**7,0**	**3069**	**93**	
<2500 grammes	76	11,3	598	88,7	2,07 [1,54-2,78]
2500-3999 grammes	140	5,8	2287	94,2	1,00
≥4000 grammes	14	7,1	184	92,9	1,24 [0,70-2,19]

### Mortalité périnatale et environnement chirurgical

58 décès périnataux sur 450 (12,9%) se sont produits parmi les nouveau-nés des opérées pour étiologies annexielles et 77 sur 1021 (7,5%) concernaient les nouveau-nés de celles opérées pour indications maternelles contre 39 des 881 (4,4%) nouveau-nés des mères opérées sur un faisceau d’arguments. Le risque létal périnatal est multiplié par près de 2 et plus de 3 et ce de manière statistiquement significative lorsque l’indication opératoire est d’origine maternelle et d’origine annexielle. Par ailleurs, 133 décès (57,3%) sont survenus chez les nouveau-nés des opérées en urgence (1187) signifiant un risque létal égal à 11,2% rattaché au caractère urgent de l’intervention contre 99 (4,9%) décès sur 2028 nouveau-nés des opérées non urgentes (OR=2,45 (1,87-3,22)). Il faut également noter que sur 215 nouveau-nés des opérées ayant été transfusées, l’on a répertorié 46 décès (21,4%) contre 200 sur 3269 (6,1%) nouveau-nés des opérées non transfusées (OR=4,17 (2,92-5,96)). La rupture utérine est grevée d’un risque létal périnatal de 40,3% contre 6,7% pour les césariennes segmentaires et corporéales prises ensemble (OR=9,45 (6,69-15,47)). Ni le type d’indication, ni le type d’anesthésie et ni le moment d’intervention ne semblent influer sur la mortalité périnatale (Tableau 6).

**Tableau 6:** mortalité périnatale en fonction de l’environnement chirurgical

Variable	Décès		Survie		OR [IC à 95%]
	n	%	n	%	
**Étiologie d’indication (n=2839)**	**201**	**7,1**	**2638**	**92,9**	
Maternelle	77	7,5	944	92,5	1,76 [1,18-2,61]
Fœtale	27	5,5	460	94,5	1,26 [0,76-2,09]
Annexielle	58	12,9	392	87,1	3,19 [2,09-4,87]
Mixte	39	4,4	842	95,6	1,00
**Type d’indications (n=3215)**	**232**	**7,2**	**2983**	**92,8**	
Élective	105	6,6	1498	93,4	1,00
Non élective	127	7,9	1485	92,1	1,22 [0,93-1,59]
**Caractère d’indications (n=3215)**	**232**	**7,2**	**2983**	**92,8**	
Urgente	133	11,2	1054	88,8	2,45 [1,87-3,22]
Non urgente	99	4,9	1929	95,1	1,00
**Type d’opérations (n=3278)**	**241**	**7,4**	**3037**	**92,6**	
Segmentaire	207	6,8	2821	93,2	1,84 [0,85-3,97]
Corporéale	7	3,8	176	96,2	1,00
Lap-Rupture utérine*	27	40,3	40	59,7	16,97 [6,90-41,71]
**Type d’anesthésie (n=3423)**	**243**	**7,1**	**3180**	**92,9**	
Générale	229	7,1	3012	92,9	1,00
Rachianesthésie	14	7,7	168	92,3	1,09 [0,62-1,92]
**Transfusion maternelle (n=3484)**	**246**	**7,1**	**3238**	**92,9**	
Oui	46	21,4	169	78,6	4,17 [2,92-5,96]
Non	200	6,1	3069	93,9	1,00
**Moment d'Intervention (n=3180)**	**223**	**7**	**2957**	**93**	
Jour	122	6,3	1808	93,7	1,00
Nuit	101	8,1	1149	91,9	1,30 [0,99-1,71]

*laparotomie

## Discussion

La recrudescence de la césarienne constatée au cours des trois dernières décennies dans les pays développés s’est accompagnée d’un bénéfice proportionnel pour le couple mère-enfant, c’est-à-dire une baisse spectaculaire de la mortalité maternelle et périnatale [23]. Pour des taux des césariennes supérieurs à 20%, la mortalité maternelle et la mortalité périnatale de 1990 à 2013 étaient passées de 7,4 à 4,8 pour 100000 naissances et de 4,4 à 2,6‰ au Danemark, de 10,3 à 3,2 pour 100000 naissances et de 4,6 à 2,3‰ en Autriche, de 10,4 à 6,1 pour 100000 naissances et de 4,5 à 2,7‰ en Angleterre et de 15,6 à 8,8 pour 100000 naissances et de 3,6 à 2,3‰ en France [8, 9, 32]. Le taux de mortalité rattaché à la césarienne y est inférieure à 0,03% pour la mère et presque nul concernant le nouveau-né [23]. Notre étude montre que la mortalité maternelle est de 390 pour 100000 naissances, taux moyen entre 2009 et 2013 et la mortalité périnatale de 46,3‰ avec des parts respectives imputables à la voie haute de 1,40% et 7,07%. Comme on le voit, comparés aux pays occidentaux, nos taux sont au moins 25 fois plus élevés concernant la mortalité maternelle globale et 50 fois pour celle imputable à la voie haute; la mortalité périnatale elle aussi est 15 fois plus élevée globalement mais près de 100 fois plus que celle imputable à la voie haute. L’évolution heureuse rapportée par de nombreux auteurs concernant la santé Mère-Enfant et l’opération césarienne diffère énormément d’un milieu à un autre en rapport avec le développement culturel, socio-économique et intellectuel des populations [24]. Nos résultats sont néanmoins proches de ceux rapportés dans la littérature concernant différents milieux de notre pays et la plupart des régions subsahariennes du continent africain ; tous ces résultats sont tout simplement l’expression d’un système sanitaire en cours de développement et d’amélioration [33,34].

### Mortalité et caractéristiques sociodémographiques des opérées

L’analyse montre que certains facteurs de risque connus dans la littérature sont liés à la morbidité fœto-maternelle péri-opératoire: la parité supérieure à 2, l’âge maternel supérieur à 35 ans. En effet, ces paramètres sont potentiellement morbides et le seraient par le biais des complications hémorragiques. La parité constitue un paramètre de risque obstétrical certain et spécialement du risque hémorragique qui s’expliquerait notamment par l’atonie utérine et les placentations vicieuses fréquentes chez les grandes multipares [35], l’âge jouant comme sujet ou conséquence. Smith, dans son étude, avait montré que l'âge avancé était associé à un degré de contraction spontanée réduite par rapport à une réponse normalisée de potassium, en d’autres termes le vieillissement est associé à une altération de la contractilité du myomètre [36] alors que la parité progresse.

### Mortalité et environnement obstétrical et chirurgical

Les césarisées hypertendues comme celles opérées sur des grossesses immatures ont présenté un risque létal significativement plus élevé. L’on serait tenté de croire qu’une grossesse immature soit couplée à un moindre risque létal maternel. La mortalité ne serait vraisemblablement liée à l’âge gestationnel mais à la raison de l’évacuation utérine par voie haute quelque soit l’âge gestationnel, c’est-à-dire que l’affection à l’origine de l’indication et son retentissement sur le bien-être maternel. Dans notre série, l’antécédent de cicatrice utérine n’avait pas influé sur la morbi-mortalité maternelle et périnatale. Son rôle comme facteur de risque est discutable car la cicatrice utérine peut servir de motif d’alerte pour une référence précoce dans un centre sécurisant mais demeure un facteur de morbidité pour les parturientes non ou mal suivies [37]. Au-delà de toute autre pathologie médico-chirurgicale et gynécologique, l’antécédent de cicatrice utérine se révèle protecteur du bien-être fœtal en ce que cet antécédent amplifie l’attention portée à la parturition et sur la sérénité des décisions obstétricales y afférentes faisant que les décisions d’extraction fœtale sont prises à temps avant toute souffrance fœtale même en l’absence du monitorage électronique intrapartale. Quant au mode d’admission, l’évacuation obstétricale influe significativement sur la mortalité périnatale (OR=1,55 (1,15-2,07)) exposant la mère à des complications péri-opératoires tout en condamnant le fœtus et/ou le nouveau-né à une forte mortalité. Ceci pourrait s’expliquer par la sous-qualification du personnel œuvrant dans les maternités périphériques, ignorant des contre-indications de la voie basse et ne référant les gestantes que très tardivement [10,11]. Dans cette étude, près de la moitié des césariennes ont été pratiquées dans un contexte d’urgence obstétricale; l’analyse statistique montre une interaction significative entre le caractère urgent de l’indication de la césarienne et la mortalité maternelle (OR=2,18 (1,22-3,88)). Ces résultats ne sont pas différents de ceux de plusieurs auteurs dans les pays développés et en développement [10, 11, 23, 38]. D’après les résultats de Foumane, la césarienne d’urgence expose la mère à l’anesthésie générale, à la non-disponibilité du bilan préopératoire, à l’infection et à une plus longue durée d’hospitalisation [38]. Si l’anesthésie péridurale présente de nombreux avantages lorsqu’elle est comparée à l’anesthésie générale au cours de la césarienne, sa réalisation dans le contexte de l’urgence reste limitée par la disponibilité d’un matériel spécifique, les compétences de l’équipe en place et le temps nécessaire à sa mise en œuvre [39]. Au total, les raisons de cette surmortalité sont celles en rapport avec les particularités de notre environnement à savoir l’absence des vraies consultations prénatales, la prolifération des maternités de fortune, la sous-qualification, le sous-équipement et le rôle prépondérant de l’ethnoculture pronataliste qui caractérise nos sociétés même en plein milieu urbain [34, 38, 40, 41]. La présente étude montre que la césarienne pratiquée dans un contexte d’urgence s’avère être un facteur de risque de mortalité périnatale (OR=2,45 (1,87-3,22)). Bien que dans notre série le risque d’asphyxie néonatale dans les césariennes d’urgence soit facilement justifiable par la plus grande fréquence des indications liées à la souffrance fœtale ou la disproportion foeto-pelvienne, il pourrait être majoré par l’utilisation plus courante en urgence d’une neuroleptanalgésie à la place d’une véritable anesthésie générale avec intubation endotrachéale ou d’une anesthésie péridurale et cela faute d’équipement et du savoir-faire alors que l’opération se fait sans assistance d’un réanimateur néonatologiste [42]. Dans nos milieux, une indication opératoire, qu’elle ait un caractère urgent ou non du point de vue obstétrical, court le même délai pour son exécution en fonction des possibilités matérielles généralement sous la responsabilité de la gestante (sa famille), pour la plupart des cas sans système de sécurité sociale. Ainsi, presque toutes les opérations se font dans les mêmes conditions et le délai souvent trop long imposé par les circonstances environnementales pour réunir le kit opératoire aggrave l’issue périnatale. Ces résultats sont similaires à ceux rapportés dans la plupart des séries africaines mais très différents des études menées dans les pays développés. A l’origine, il s’agit des mêmes causes déjà et plusieurs fois évoquées dans cette discussion et qui découlent du sous-développement global de la société [10, 23, 41, 43].

## Conclusion

Cette étude montre que la césarienne dans nos conditions de travail est couplée à une forte mortalité maternelle et périnatale. Les facteurs de risque identifiés (l’âge maternel avancé, la grande multiparité, l’absence de suivi prénatal, le terme précoce de l’évacuation de la grossesse, l’évacuation obstétricale, le caractère urgent de l’indication, la rupture utérine) sont en grande partie évitables, surtout à tort ou à raison imputés à l’opération au profit des circonstances souvent irrationnelles de sa pratique. L’imputabilité de la morbidité et mortalité maternelles et périnatales rattachées à la voie haute requiert d’urgence une profonde réflexion conduisant au vrai discernement des raisons primaires qui aboutiront à révolutionner la pratique obstétricale en la rendant plus rationnelle et moins délétère.

### Etat des connaissances actuelles sur le sujet

- La recrudescence de la césarienne dans les pays développés s’est accompagnée d’un bénéfice proportionnel pour le couple mère-enfant;
- En Afrique subsaharienne, la césarienne reste une opération qui s’accompagne d’une forte morbidité et mortalité maternelles et périnatales dans nos conditions de travail;
- Aucune donnée récente disponible sur ce sujet à Lubumbashi.

### Contribution de notre étude à la connaissance

- Première étude multicentrique dans notre contexte, à Lubumbashi, République Démocratique du Congo;
- L'étude proposée est la première étude analytique globale permettant de répertorier les facteurs de risque de la mortalité maternelle et périnatale (ce qui est important pour en déduire des perspectives d'action);
- L'étude proposée est la première étude permettant d’évaluer le pronostic maternel et périnatal dans notre contexte.
